# Intercropping Salt-Sensitive *Lactuca sativa* L. and Salt-Tolerant *Salsola soda* L. in a Saline Hydroponic Medium: An Agronomic and Physiological Assessment

**DOI:** 10.3390/plants11212924

**Published:** 2022-10-30

**Authors:** Giulia Atzori, Werther Guidi Nissim, Stefano Mancuso, Emily Palm

**Affiliations:** 1 Dipartimento di Scienze e Tecnologie Agrarie, Alimentari, Ambientali e Forestali (DAGRI), University of Florence, Viale delle Idee 30, 50019 Sesto Fiorentino, Italy; 2 Department of Biotechnology and Biosciences, University of Milano-Bicocca, Piazza della Scienza 3, 20126 Milano, Italy; 3 Fondazione Futuro delle Città—FFC, 50125 Firenze, Italy

**Keywords:** biosaline agriculture, seawater irrigation, obligate halophyte, glycophyte, photosynthesis, nutritional profile

## Abstract

Competition for freshwater is increasing, with a growing population and the effects of climate change limiting its availability. In this experiment, *Lactuca sativa* plants were grown hydroponically with or without a 15% share of seawater (12 dS m^−1^) alone or intercropped with *Salsola soda* to demonstrate if *L. sativa* benefits from sodium removal by its halophyte companion. Contrary to the hypothesis, saline-grown *L. sativa* plants demonstrated reduced growth compared to the control plants regardless of the presence or absence of *S. soda*. Both limitations in CO_2_ supply and photosystem efficiency may have decreased CO_2_ assimilation rates and growth in *L. sativa* plants grown in the seawater-amended solutions. Surprisingly, leaf pigment concentrations increased in salt-treated *L. sativa* plants, and most notably among those intercropped with *S. soda*, suggesting that intercropping may have led to shade-induced increases in chlorophyll pigments. Furthermore, increased levels of proline indicate that salt-treated *L. sativa* plants were experiencing stress. In contrast, *S. soda* produced greater biomass in saline conditions than in control conditions. The mineral element, carbohydrate, protein, polyphenol and nitrate profiles of both species differed in their response to salinity. In particular, salt-sensitive *L. sativa* plants had greater accumulations of Fe, Ca, P, total phenolic compounds and nitrates under saline conditions than salt-tolerant *S. soda*. The obtained results suggest that intercropping salt-sensitive *L. sativa* with *S. soda* in a hydroponic system did not ameliorate the growing conditions of the salt-sensitive species as was hypothesized and may have exacerbated the abiotic stress by increasing competition for limited resources such as light. In contrast, the saline medium induced an improvement in the nutritional profile of *S. soda*. These results demonstrate an upper limit of the seawater share and planting density that can be used in saline agriculture when intercropping *S. soda* plants with other salt-sensitive crops.

## 1. Introduction

Alternative sources of irrigation for food production are needed to meet the demand created by an ever-growing population and dwindling freshwater resources caused by climate change. The irrigation of conventional crops currently accounts for roughly 70% of total freshwater use [1], much of it as extracted groundwater that is poorly managed [2,3]. To reduce the water footprint of cultivated crops, several alternatives are being investigated, including the partial use of seawater in irrigation and the cultivation of salt-tolerant species or halophytes [4].

Exposure to pure seawater is lethal to most plant species as excess concentrations of sodium (Na) in plant tissues causes an imbalance in the K^+^/Na^+^ ratio [5] that results in reductions in growth through damage to the photosynthetic machinery [6,7] and cell membrane integrity [8]. Halophytes, plant species that are moderately or highly tolerant to saline conditions, often possess mechanisms to successfully exclude Na from plant tissues, as in *Brassica napus* L. [9], or sequester Na in specific structures as in the leaf bladder cells of *Atriplex canescens* (Pursh) Nutt [7], and do not show the symptoms of Na toxicity that are common among salt-sensitive species or glycophytes. Crops that can tolerate species-specific degrees of salinity can be found among the edible halophytes, species which have naturally adapted to saline environments [10]. They are capable of growth and reproduction at soil salinities greater than 200 mM NaCl, which corresponds roughly to ~40% of seawater salinity [11].

The interest in these species is timely, as their domestication could allow for the exploitation of more available brackish water and seawater sources for sustainable food production in salt-rich environments [12,13]. There is increasing interest in the use of intercropping in saline agriculture by growing salt-sensitive and salt-tolerant plant species together using irrigation mixtures of freshwater and seawater [14]. Since a number of these salt-tolerant species are characterized by Na uptake activity, they would permit soil phytodesalination by exploiting a halophyte’s natural capacity to absorb toxic Na^+^ ions, while simultaneously increasing the amount of water available for crop cultivation and lowering the freshwater footprint of individual crops. The efficiency of Na uptake has been evaluated in different trials for multiple species [15,16,17]. Similarly, the study of crop rotation and intercropping of salt-sensitive and salt-resistant species in saline environments has been successfully explored with the majority of experiments concluding that the salt-sensitive species can benefit from the phytodesalination performed by the salt removing species [18,19,20,21]. Such trials are mainly performed under soil conditions, yet soilless systems are known to achieve better water use efficiency compared to field conditions [4,5,6,7,8,9,10,11,12,13,14,15,16,17,18,19,20,21,22], and growing crops in hydroponics under saline conditions has already been successfully tested. However, to-date, the literature still lacks data from intercropping salt-sensitive and salt-tolerant crops in hydroponic conditions. The present trial assessed whether intercropping under hydroponic saline conditions is beneficial for a salt-sensitive species due to Na removal by the halophyte companion crop. The glycophyte *Lactuca sativa* L. was intercropped with the edible halophyte *Salsola soda* L. (hereafter referred to as *L. sativa* and *S. soda*, respectively) in a hydroponic culture with an electrical conductivity (EC) of 12 dS m^−1^ established with the addition of a 15% share of seawater. An earlier study demonstrated that an EC of 12 dS m^−1^ from seawater significantly reduced the dry biomass of *L. sativa* [23], indicating an upper limit of its tolerance to shares of seawater in hydroponic culture. The goal of the present study was to assess whether *S. soda*, which has shown desalination potential, could alleviate the negative effect of such a high level of conductivity and sodium toxicity in an otherwise salt-sensitive plant species, *L. sativa*. It was hypothesized that the 1:50 ratio of *L. sativa*: *S. soda* would reduce the amount of Na in the solution through absorption by *S. soda* plants and prevent Na-induced reductions in *L. sativa* growth.

## 2. Results and Discussion

### 2.1. Different Salinity-Induced Response among the Tested Crops

Overall, there was no positive effect on any of the growth or physiological parameters measured in *L. sativa* by intercropping it with *S. soda* under saline conditions. Furthermore, though the pH of the growth solutions remained stable over the course of the trial, the EC of the saline solutions increased, more so when *L. sativa* was intercropped with *S. soda* (Table 1).

**Figure 1:**
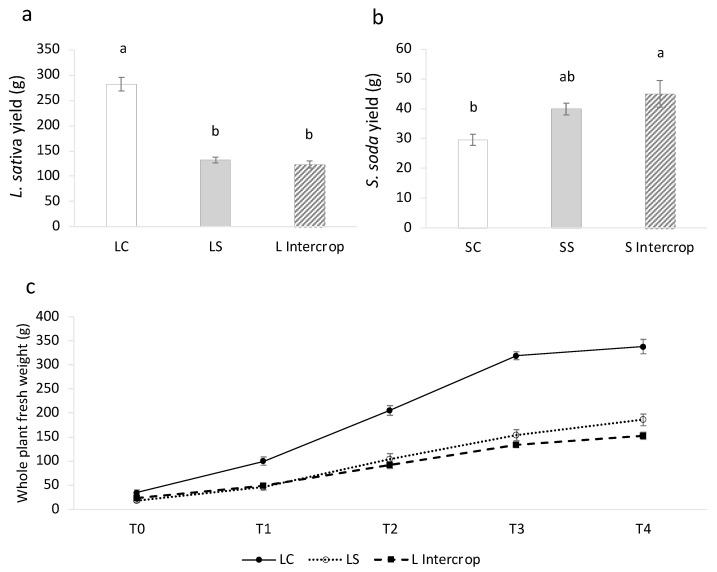
Edible *L. sativa* (**a**) and *S. soda* (**b**) yield expressed as the average shoot fresh weight and weekly whole plant fresh weights (**c**) in grams. All data points are means ± SEM. Different lowercase letters indicate significant differences between treatment groups within the same species (*p* < 0.05) based on one-way ANOVA.

As expected, *S. soda,* as an obligate halophyte, performed better under saline conditions as compared to the control grown *S. soda* plants (Figure 1). A slightly increased yield (edible fresh shoot tissue) is observed in intercropped plants compared to SS plants, despite the same EC in the two treatments. By contrast, the edible yield of *L. sativa* was significantly reduced in saline conditions regardless of intercropping, suggesting that the removal of Na performed by the halophyte companion was not sufficient to improve the quality of the growing medium. A summary of additional growth parameters of the two species is shown in Table 2.

In general, *L. sativa* showed significant reductions in all investigated parameters in saline conditions compared to the control, regardless of the presence or absence of *S. soda*, with the exception of root fresh weight. On the contrary, *S. soda* showed both an increased yield (Figure 1b) and an increased development of the root system (Table 2) when treated with salt. Weekly measurements of *L. sativa* whole plant fresh weight (Figure 1c) demonstrated that saline conditions reduced relative growth rates as compared to control grown plants from the beginning of the trial (T1).

### 2.2. Physiological Response Suggests No Benefit Due to Intercropping under Saline Conditions

**Figure 2:**
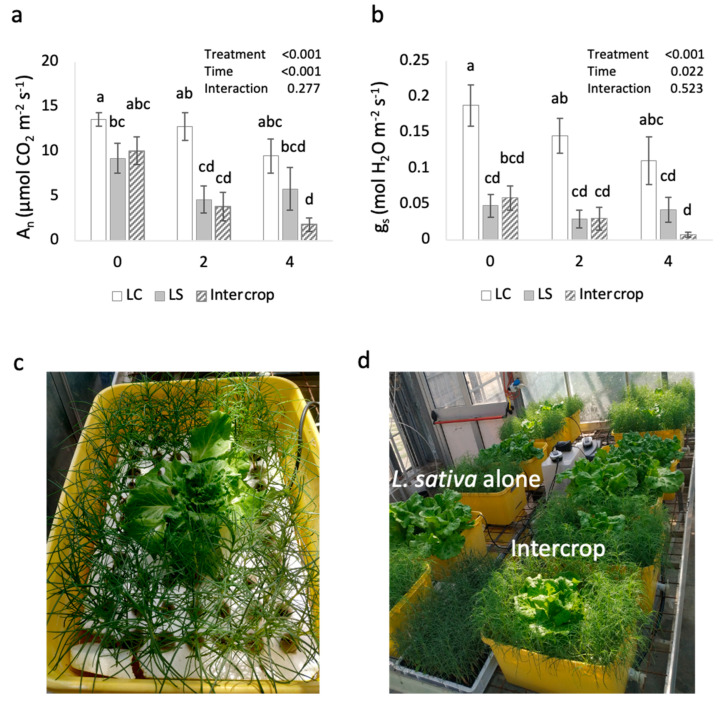
Gas exchange parameters, carbon assimilation rate (**a**) and stomatal conductance rate (**b**), at three time points (0, 2 and 4 weeks) and visual observations of intercropped *L. sativa* with *S. soda* at 1 week (**c**) and 4 weeks (**d**). Columns represent the mean of *n* = 6 with SEM. Different lowercase letters indicate significant differences between treatment groups over time (*p* < 0.05) based on a two-way ANOVA and a post-hoc Tukey HSD.

Gas exchange and photosystem parameters of fluorescence and pigments were measured at the beginning, middle and end of the trial in *L. sativa* plants and showed no benefit when grown together with *S. soda* under saline conditions. The carbon assimilation (A_N_) and stomatal conductance (g_s_) rates (Figure 2a,b) clearly show that intercropping with *S. soda* under saline conditions further reduced gas exchange parameters significantly over time, even more so than in *L. sativa* grown alone in 15% seawater. By week 4, L intercrop values of A_N_ and g_s_ were 1.78 and 0.007, respectively, compared to 9.49 and 0.111, and 5.79 and 0.042 of the LC and LS plants, respectively.

Reductions in A_N_ and g_s_ are expected under saline conditions in salt-sensitive species such as *L. sativa*. Osmotic stress, and increased Na^+^ both induce stomatal closure leading to reduced CO_2_ assimilation. The gas exchange data in Figure 2 does not provide support for the initial hypothesis, instead indicating that intercropped *L. sativa* plants were experiencing salt stress, and that the *S. soda* plants did not provide phytodesalinizing benefits. The declines in gas exchange parameters were not explained by the data regarding photosystem II function and capacity, fluorescence and pigments, respectively (Table 3). Both the maximum fluorescence and the efficiency of open reaction centers changed little over time and between the treatment groups, suggesting that there was no direct, negative effect due to salinity on the function of photosystem II. This is further supported by the slight increase in chlorophyll a and b, as well as the accessory pigments, carotenoids, in salt-treated lettuce and intercropped *L. sativa* plants.

Salt stress generally leads to degradation of chlorophyll pigments as increased concentrations of Na^+^ in the growing media upset the internal K^+^/Na^+^ homeostasis and stimulate ROS production [6,7]. Here we found that pigment concentrations increased, especially in the intercropped *L. sativa*, suggesting that these plants were likely also suffering from shade stress, increasing chlorophyll and carotenoid concentrations to compensate [24]. Visual observations such as the photos in Figure 2c,d indicate this as well, with the *S. soda* plants crowding the *L. sativa* by week 4 of the treatment period (Figure 2d), relative to the first week of the experiment (Figure 2c). In addition, to facilitate gas exchange and fluorescence measurements, *S. soda* plants immediately surrounding the *L. sativa* had to be repositioned and pushed aside to make space for the measurement chamber of the instrument. The possibility that shade stress was a factor is further supported by the values for photosystem II efficiency (ΦPSII) and non-photochemical quenching which demonstrate that the leaves were adapting to low light environments and could not take advantage of high intensity light supplied during the measurement. The dry weight of *L. sativa* plants grown with *S. soda* was lower than when grown alone suggesting that there may have been increased competition for resources, including nutrients and light (shading), in addition to the EC of the solutions. EC values of the saline and intercropped bins were equal (roughly 12 dS m^−1^) at the start of the trial and after changing the solutions at the midpoint. However, they increased dramatically after two weeks to 16.3 dS m^−1^ in the intercrop treatment. A similar increase was observed when *S. soda* was grown alone in saline conditions, indicating that *S. soda* did not reduce salt content of the growth solutions as was hypothesized. These results are in direct contrast to those obtained by Colla et al. [18] in which *S. soda* was intercropped with pepper plants at a ratio of 60: 1. In that case, the experiment was performed in a 1:2 volume ratio of peat:pumice which may have buffered the Na toxicity better than hydroponic culture and the pepper plants may have been more successful than *L. sativa* in outcompeting *S. soda* for light and other limited nutrient resources. To confirm this idea, a follow-up experiment with *L. sativa* and *S. soda* should be performed in soil with the same species and the application of EC 12 dS m^−1^ irrigation water to evaluate the buffering effect of the soil in intercropping setups with these two species. Additionally, the EC of the irrigation solutions used here were in line with previously performed experiments exploring the threshold of *L. sativa* in saline agriculture [23]. In that case, a 15% share of seawater and EC 12 dS m^−1^ in the hydroponic solution was already found to have negative effects on *L. sativa* growth. The present study sought to investigate the potential of a halophyte companion plant to reduce the EC of the solution and improve yield under these specific conditions. However, the share of seawater used in this trial exceeded that of Colla et al. 2006 [18] in which a positive effect of phytodesalinization and an improvement in the yield of *Capsicum annuum* L. was observed. To eliminate the possible effect due to the growth media (i.e., soil versus hydroponics), similar seawater shares to that of Colla et al. should be attempted in hydroponic culture. Furthermore, the results from this experiment indicate that it may be necessary to explore more complex intercropping techniques for hydroponic culture, using halophyte species as a pre-biofilter, rather than cultivating multiple species in the same physical space to avoid confounding abiotic factors such as direct competition for light.

### 2.3. Nutritional Profile of L. sativa and S. soda

Table 4 reports the mineral element accumulation in the leaves of the two crops: as found with growth parameters, the two species showed diverse accumulation patterns. Notably, *L. sativa* showed an increased concentration of P, Fe, Mg, Zn and Na in the Intercrop treatment relative to plants grown in the control solution, and a significant increase in Ca, Fe, Mg and P compared to individuals grown alone in the saline solution. Such enhanced accumulation represents an important achievement when the fact that the group of mineral elements most commonly lacking in human diets includes the above-mentioned elements [25] is taken into consideration. *S. soda* plants accumulated mineral elements to a lesser extent in saline versus non-saline conditions (i.e., Zn, Na and Mo), suggesting that *L. sativa* did adjust the allocation of elements as a consequence of salt stress that was more or less unperceived by *S. soda* plants.

As reported in Table 5, *L. sativa* did not show any significant differences in the production of carbohydrates and proteins among the treatments used, whereas among *S. soda* plants, a significant increase for both compounds was observed in saline conditions as compared to the control.

The concentration of total phenolic compounds (TPC) increased in intercropped *L. sativa* compared to both other treatments, whereas it decreased with increasing salinity in *S. soda* plants (data reported in Table 5), has already been found for other halophyte species [23]. Polyphenols are among the secondary metabolites whose concentration is modulated by plants as a function of osmotic regulation, but the specific role of their concentration change is still not fully understood [26]. In terms of nitrate concentrations in leaves at harvest (Table 5), the two species displayed contrasting responses: while *L. sativa* increased its nitrate concentration under saline conditions, *S. soda* significantly reduced it. The halophyte behavior in this case is consistent with that of other halophytes [27] and is justified by the competition between compartmentalizing sodium or nitrates in the vacuole. From a nutritional perspective, it is also a target for leaf vegetables to reduce the nitrate content in leaves. *L. sativa* instead showed a remarkable increase in its nitrate concentration, though it is still below the recommended maximum daily intake values.

## 3. Materials and Methods

### 3.1. Plant Material, Growth Conditions and Experimental Design

The trial was carried out in 2021 at the greenhouse facilities of the Department of Agricultural, Food, Environmental and Forestry Sciences and Technologies (DAGRI)-University of Florence, Italy, with *Lactuca sativa* L. (*L. sativa*) and *Salsola soda* L. (*S. soda*) plants. A hydroponic system was set up with plants floating on polystyrene layers in 12 plastic containers (25 L volume) containing continuously aerated Hoagland solution, in a modified version of the protocol described in Colla et al. 2006 [18]. Here, the same modified Hoagland solution was used, but slight adjustments were made as to the application of salt in the saline treatment and the ratio of glycophyte:halophyte, as described below. The seawater used was collected at Marina di Pisa (Italy) one week before the beginning of the experiment and stored at 4 °C. A salinity level characterized by an EC of 12 dS m^−1^ was chosen for the salt treatment based on a previous study that found a decrease in dry biomass production in *L. sativa* at that EC relative to the two lower levels of EC tested (i.e., EC 5.5 dS m^−1^ and control conditions) [23].

The two species were assigned in a completely randomized design to the two treatment solutions as follows:

LC: *L. sativa* plants grown on modified Hoagland solution (2 plastic containers with 3 plants each, *n* = 6);
LS: *L. sativa* plants grown on modified Hoagland solution with a 15% share of seawater, final EC of 12 dS m^−1^ (2 plastic containers with 3 plants each, *n* = 6);
SC: *S. soda* plants grown on modified Hoagland solution (1 plastic container with 56 plants, *n* = 56);
SS: *S. soda* plants grown on modified Hoagland solution with a 15% share of seawater, final EC of 12 dS m^−1^ (1 plastic container with 56 plants, *n* = 56);
Intercrop: *L. sativa* and *S. soda* plants intercropped (1 *L. sativa*: 50 *S. soda;* ratio chosen based on the results of a previous publication assessing successful desalination by intercropping 1 pepper plant: 60 *S. soda* in a soil-based media; [18]) on modified Hoagland solution with a 15% share of seawater, final EC of 12 dS m^−1^ (6 plastic containers with 1 *L. sativa* and 50 *S. soda* plants each, *n* = 6 for lettuce and *n* = 300 for *S. soda*).

**Figure 3:**
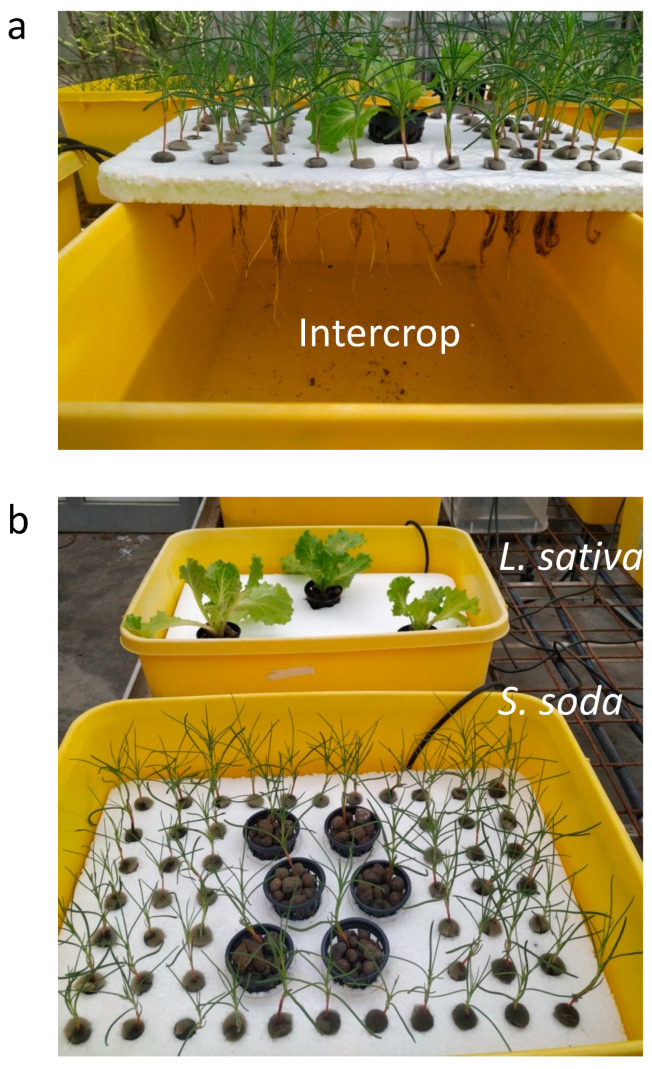
The experimental set-up for all three treatment groups at start of the trial: (**a**) intercrop and (**b**) *L. sativa* alone (**top**) and *S. soda* alone (**bottom**).

*L. sativa* and *S. soda* plants were obtained from a local nursery, transplanted into 5 cm mesh pots filled with expanded clay and allowed to adapt to hydroponic conditions for 7 days. The plants were then transferred along with their mesh pots to polystyrene layers floating on the nutrient solution as shown in Figure 3. *S. soda* plants were held in place with foam plugs, with the exception of six plants that were contained in mesh pots and centrally located selected for measurements in the SC and SS treatments. Plants of treatments LS, SS and Intercrop were gradually acclimatized to salinity by increasing the seawater concentration by 5% every 2–3 days until reaching the final concentration on 21 May, which represents the starting day of the experiment.

The experiment lasted 4 weeks (21 May to 18 June) and was designed to encompass a complete indoor *L. sativa* crop cycle (approx. 30 days). Throughout the trial, plants were maintained at a relative humidity ranging from 40 to 55% and natural sunlight with the light intensity reaching a maximum of 700 μmol m^−2^ s^−1^ on clear days. Daytime and nighttime temperatures averaged 28 °C and 18 °C, respectively, over the experimental period, ranging between 22–32 °C during the day and 11–19 °C during the night. No supplementary lamps were used during the trial. The daylength increased from 14.83 h to 15.45 h by the end of the trial. Samples from the nutrient solution were collected once a week, and pH and EC were measured by a combined pH and electroconductivity meter (pH meter PHM 210 Meter Lab, Radiometer Analytical). Nutrient solutions were replaced at the midpoint of the trial (2 weeks).

### 3.2. Growth and Biomass Yield Assessment

Biomass growth of each crop was determined by weighing all plants weekly along with their pots. For both SC and SS treatments, 6 plants were selected to regularly monitor growth during the trial. After plant sampling, empty pots and impermeable expanded clay weight was subtracted from the previous weights, thus obtaining the exact entire fresh weights of individual plants. At harvest time, samples from fresh leaves from both species were collected, frozen into liquid nitrogen and then stored at −80 °C for further analysis of pigments, carbohydrate, protein, proline and polyphenol concentrations. Plants were divided into shoots and roots and weighed individually to obtain fresh weight. All samples were then oven-dried at 70 °C until constant weight and dry biomass was determined.

### 3.3. Physiological Parameters: Photosynthesis, Fluorescence, and Pigments

The effect of seawater cultivation on photosynthetic machinery was evaluated 3 times on *L. sativa* plants during the trial with the Licor 6400XT open system (Licor Biosciences, Lincoln, Nebraska USA). Light-adapted measurements of gas exchange (A_N_ and g_s_) and fluorescence (F’v/F’m and ΦPSII) were collected on fully expanded leaves of *L. sativa*. Conditions in the chamber were set at 420 µmol CO_2_ m^−2^ s^−1^, 1000 µmol m^−2^ s^−1^ PAR, 40–60% RH and 24 °C. Dark-adapted fluorescence (Fv/Fm) values were acquired by covering a leaf in aluminum foil the night before measurements and collecting Fo and Fm values before the application of light. NPQ was calculated from Fm and F’m [28]. Pigment concentrations were assessed from leaf tissue samples collected at the time of gas exchange and fluorescence measurements for *L. sativa* and at the end of the trial for *S. soda*. Chlorophyll a and b and total carotenoids were extracted with methanol following the protocol described by Wellburn [29] from leaf samples, on 6 replicates per treatment. Absorbances were read with a Tecan Infinite 2000 spectrophotometer (Tecan; Männedorf, Switzerland). Concentrations of pigments were calculated following the equations in Wellburn [29] and expressed as µg g^−1^ fresh weight of leaf tissue.

### 3.4. Concentration of Mineral Elements in Plant Tissues

Oven-dried leaf ground samples (6 replicates per treatment) were analyzed for the determination of N and C through an elemental analyzer Thermo Finnigan Flash 1112 Series EA (Thermo Finnigan Italia S.p.A., Milan, Italy). The dried samples were then mineralized with the Aqua Regia extraction method based on acid digestion in a CEM microwave using the same procedure and settings described in Guidi Nissim et al., 2019 and diluted samples were used to determine P, K, Ca, Fe, Mg, Mn, Cu, Zn, Na and Mo concentrations by means of ICP OES (Inductively Coupled Plasma—Optical Emission Spectrometer) Thermofisher Iris Intrepid II (Thermo Fisher Scientific, Waltham, MA, USA) based on Atomic Emission Spectroscopy.

### 3.5. Concentration of Carbohydrates, Proteins, Polyphenols, Proline and Nitrates in Plants Edible Leaves

The concentration of carbohydrates, proteins, polyphenols, proline and nitrates was measured on leaf samples collected at the end of the trial with 6 replicates per treatment for both species. All absorbances for the following methods were read with a Tecan Infinite 2000 spectrophotometer (Tecan; Männedorf, Switzerland) at the specified wavelengths. Leaf tissue carbohydrates were measured following the Dubois method [30]. Briefly, samples are first extracted in water and then left to react with a 5% *w*/*v* phenol solution and sulfuric acid for 10 min. Samples were cooled in an ice bath before reading absorbance values at 488 nm. Protein concentrations were measured using the BioRad Protein Assay that is based on the Bradford dye method (BioRad Product No. 500-0006) and a standard curve using bovine serum albumin. After a preliminary extraction of lyophilized samples in water, samples react with the Bio Rad Bradford Dye. Samples and the dye were pipetted into wells of a microspectrophotometer plate, and sample absorbance values read at 595 nm. Total phenolic compounds (TPC) concentration was measured using the Folin–Ciocalteu reagent according to a common standard method [31] samples are extracted in methanol and then transferred to a reaction with the F-C reagent and 700 mM Na_2_CO_3_. After an incubation period of 2 h, absorbance of each sample was read at 765 nm. Proline concentrations were evaluated following the protocol described by other researchers [32]. Samples are extracted in ethanol, and then transferred to a reaction mix of ninhydrin 1% (*w*/*v*), acetic acid 60% (*v*/*v*) and ethanol 20% (*v*/*v*). Samples with reaction mixtures were incubated at 95 °C for 20 min, cooled to room temperature and then read at 520 nm. Following the method described by Cataldo et al. [33], nitrates were extracted from dry samples in water and reacted with sulfur salicylic acid and a solution 1.5 N of NaOH. Samples were then cooled to room temperature before reading the absorbance at 410 nm.

### 3.6. Statistical Analyses

Statistical analyses were conducted using GraphPad Prism v.6 for Windows (Graphpad Software; San Diego, California, USA). According to the different datasets, one-way ANOVA or repeated measures two-way ANOVA analysis of variance was used to assess significant differences between treatments for the individual species. In particular, growth parameters, mineral element, carbohydrate, protein, polyphenol and nitrate concentrations were analyzed through one-way ANOVA and Tukey’s Test Multiple Comparisons (LC vs. LS vs. L intercrop and SC vs. SS vs. S intercrop) or unpaired *t*-test (SC vs. SS) at a significance level of *p* ≤ 0.05. A repeated measures two-way ANOVA was instead used to evaluate main factors (solution/intercropping treatment and time) and interaction effects for all physiological parameters that were evaluated at three time points during the trial. A post-hoc Tukey HSD was applied to test for differences between treatment combinations with a significance level of *p* ≤ 0.05.

## 4. Conclusions

*S. soda* did not improve the growth of *L. sativa* when intercropped together in saline conditions of 12 dS m^−1^ with seawater in hydroponic culture relative to *L. sativa* grown alone in EC 12 dS m^−1^, as was hypothesized. The Na uptake capacity of the obligate *S. soda* was not optimized in this setup and may have instead exacerbated the Na-induced stress in *L. sativa* through competition for other limited resources, in particular, light. However, the positive effects of intercropping on TPC, Ca, Fe and P suggest that some qualitative traits of *L. sativa* may be improved when grown along with *S. soda* in hydroponics using saline water. Other arrangements should be explored such as repeating the intercropping at lower EC values, using soil as the growth medium or using the halophyte as a biofilter to remove excess Na from a solution before application to the salt-sensitive partner. This study also demonstrates the improved performance of the halophyte *S. soda* in saline conditions compared to freshwater cultivation. The severe effects due to climate change include a reduction in the availability of freshwater for agriculture. Halophyte species such as *S. soda* deserve special attention as they may represent an important integration in the human diet that can be achieved while preserving natural resources. Though already considered a staple in some areas of the Mediterranean, it would be interesting to study whether such appreciation of *S. soda* would further increase on a global scale if consumers were aware of its lower pressure on natural resources compared to similar products.

## Figures and Tables

**Table 1:** Changes in solution pH and electrical conductivity (EC) over the 4-week trial with a complete replacement of the solutions at 2 weeks. Values are means ± SEM.

	Change in pH				Change in EC (dS m^−1^)			
	Starting pH	End of First Half	Starting pH	End of Second Half	Starting EC	End of First Half	Starting EC	End of Second Half
LC	6.2	6.1 ± 0.20	6.2	6.4 ± 0.10	2.3	3.5 ± 0.17	2.6	3.3 ± 0.09
LS	6.4	6.1 ± 0.00	6.4	6.2 ± 0.00	12.0	15 ± 0.19	11.6	15.4 ± 1.01
Intercrop	6.3	6.3 ± 0.04	6.3	6.1 ± 0.03	12.0	16.3 ± 0.33	11.8	16.3 ± 0.25
SC	6.1	6.1 ± 0.00	6.1	6.1 ± 0.00	2.3	4.2 ± 0.00	2.7	3.1 ± 0.00
SS	6.3	6.2 ± 0.00	6.3	5.9 ± 0.00	12.0	15.4 ± 0.00	11.1	14.3 ± 0.00

**Table 2:** Growth of *L. sativa* and *S. soda* plants in terms of both fresh and dry biomass at the end of the 30-day trial.

Treatments	Total Plant FW	Total Plant DW	Root FW	Root DW	Root:Shoot Ratio
	g	g	g	g	
LC	323.4 ± 15.1 a	11.9 ± 0.7 a	41.2 ± 2.5	2.2 ± 0.3 a	0.22 ± 0.02
LS	173.8 ± 7.99 b	8.998 ± 0.4 b	41.8 ± 0.6	1.7 ± 0.1 ab	0.24 ± 0.01
L intercrop	155.2 ± 8.7 b	8.6 ± 0.4 b	31.98 ± 2.1	1.4 ± 0.04 b	0.19 ± 0.01
SC	31.3 ± 2.2	2.4 ± 0.1	1.7 ± 0.5 b	0.1 ± 0.03 b	0.1 ± 0.01
SS	44.1 ± 1.5	3.1 ± 0.2	4.2 ± 1.1 a	0.2 ± 0.02 a	0.08 ± 0.02
S intercrop	47.5 ± 4.5	3.3 ± 0.3	2.4 ± 0.2 ab	0.1 ± 0.01 b	0.07 ± 0.01

Values are means ± SEM of 6 replicates per treatments LC, LS, L intercrop, SC, SS, and S intercrop. Different letters in the same column and for the same crop indicate a significant difference at *p* < 0.05 (Tukey HSD test).

**Table 3:** Physiological parameters investigated in *L. sativa* (Time 0, 2 and 4) and *S. soda* (Time 4).

Parameters	Treatment Group	Time			Two-Way ANOVA Results	
		0 Weeks	2 Weeks	4 Weeks		
Fv/Fm	LC	0.82 ± 0.002 a	0.82 ± 0.001 a	0.80 ± 0.005 ab	Treatment	0.473
	LS	0.82 ± 0.001 ab	0.82 ± 0.003 a	0.81 ± 0.003 ab	Time	<0.001
	L Intercrop	0.82 ± 0.003 a	0.82 ± 0.004 ab	0.79 ± 0.017 b	Interaction	0.273
F’v/F’m	LC	0.45 ± 0.008 ab	0.49 ± 0.011 a	0.46 ± 0.013 ab	Treatment	<0.001
	LS	0.41 ± 0.028 abc	0.37 ± 0.021 c	0.41 ± 0.011 abc	Time	0.454
	L Intercrop	0.42 ± 0.025 abc	0.37 ± 0.018 c	0.39 ± 0.011 bc	Interaction	0.095
ΦPSII	LC	0.21 ± 0.010 ab	0.24 ± 0.019 a	0.20 ± 0.018 ab	Treatment	0.008
	LS	0.20 ± 0.024 ab	0.17 ± 0.016 ab	0.18 ± 0.030 ab	Time	0.146
	L Intercrop	0.20 ± 0.025 ab	0.15 ± 0.17 ab	0.13 ± 0.013 b	Interaction	0.182
NPQ	LC	3.01 ± 0.083 a	2.26 ± 0.134 c	2.24 ± 0.113 c	Treatment	0.010
	LS	2.96 ± 0.167 a	2.78 ± 0.079 ab	2.43 ± 0.094 bc	Time	<0.001
	L Intercrop	3.00 ± 0.96 a	2.79 ± 0.077 ab	2.45 ± 0.036 bc	Interaction	0.07
Chl a	LC	210.0 ± 9.9 a	238.3 ± 21.1 a	204.7 ± 18.4 a	Treatment	0.110
µg g^−1^ FW	LS	235.9 ± 16.1 a	266.3 ± 8.1 a	220.0 ± 25.3 a	Time	0.007
	L Intercrop	221.1 ± 10.7 a	286.1 ± 17.3 a	235.9 ± 24.8 a	Interaction	0.817
	SC			276.5 ± 20.0		
	SS			252.2 ± 15.7		
Chl b	LC	60.2 ± 4.8 a	110.4 ± 40.4 a	58.1 ± 2.2 a	Treatment	0.531
µg g^−1^ FW	LS	57.1 ± 2.8 a	67.5 ± 3.6 a	64.8 ± 5.8 a	Time	0.134
	L Intercrop	69.1 ± 7.2 a	71.9 ± 4.9 a	67.4 ± 4.4 a	Interaction	0.315
	SC			55.7 ± 1.7		
	SS			55.5 ± 1.7		
Carotenoids	LC	65.1 ± 3.6 ab	64.7 ± 8.4 ab	60.7 ± 6.5 b	Treatment	0.004
µg g^−1^ FW	LS	80.0 ± 6.1 ab	85.4 ± 2.9 ab	66.3 ± 8.7 ab	Time	0.048
	L Intercrop	71.8 ± 3.7 ab	91.4 ± 5.9 a	76.8 ± 6.7 ab	Interaction	0.358
	SC			81.6 ± 5.8		
	SS			70.4 ± 4.7		

Photosystem II fluorescence parameters and leaf photosynthetic pigments of lettuce at three time points (0, 2 and 4 weeks) and pigments in *S. soda* at 4 weeks. Fv/Fm is maximum photosynthetic capacity, F’v/F’m is the efficiency of open reaction centers, ϕPSII represents the efficiency of photosystem II, and NPQ, non-photochemical quenching. Values are the mean ± SEM of 6 replicates per treatments LC, LS, L intercrop, SC and SS. Different letters in the same column and for the same crop indicate a significant difference at *p* < 0.05 (Tukey HSD test). Main factor effects (treatment and time) as well the interaction effect from the two-way ANOVA are reported for each parameter.

**Table 4:** Mineral element accumulation in the edible leaves.

**Treatments**	**P**	**K**	**Ca**	**Fe**	**Mg**
	**ppm**	**ppm**	**ppm**	**ppm**	**ppm**
LC	6841.6 ± 252.8 b	54,420.8 ± 3141.8 a	8028.3 ± 155.81 a	98.6 ± 5.4 a	2401.8 ± 119.8 b
LS	7176.5 ± 38.9 b	42,996 ± 454.8 b	4851.1 ± 87.2 c	65.3 ± 2.8 b	2606.5 ± 78.8 ab
L Intercrop	7773.5 ± 74.7 a	41,608.3 ± 1265.1 b	5537.8 ± 235.2 b	107.9 ± 23.2 a	3063.5 ± 276.1 a
SC	12334.7 ± 430.6 a	60,710.7 ± 1584.7 a	7541.3 ± 351.4 a	64.8 ± 1.8 a	2785.3 ± 162.4 a
SS	8438.3 ± 211 b	43,029 ± 565.5 b	3891 ± 195.1 b	70.3 ± 2.9 a	2856.5 ± 167 a
S Intercrop	8705.4 ± 567.6 b	44,459.5 ± 688.3 b	3558.8 ± 253.1 b	69.7 ± 2.3 a	2469.4 ± 331.2 a
**Treatments**	**Mn**	**Cu**	**Zn**	**Na**	**Mo**
	**ppm**	**ppm**	**ppm**	**ppm**	**ppm**
LC	79.9 ± 2.3 b	12.6 ± 0.4 a	57.4 ± 3.2 b	1010.9 ± 82.7 b	0.9 ± 0.05 a
LS	155.1 ± 7.7 a	12.7 ± 0.1 a	90.5 ± 4.5 a	20,739.3 ± 529.7 a	0.5 ± 0.1 b
L Intercrop	88.7 ± 3.7 b	14.1 ± 0.7 a	84.8 ± 8.4 a	24,925 ± 1686.6 a	0.6 ± 0.02 b
SC	55.1 ± 3.6 a	14.6 ± 0.3 a	47.9 ± 2.2 b	13,820.6 ± 481.7 b	1.8 ± 0.1 b
SS	61 ± 4.7 a	14.9 ± 0.7 a	58.6 ± 3.3 ab	44,936 ± 804.7 a	2.3 ± 0.1 a
S Intercrop	73 ± 6.9 a	18.7 ± 2.4 a	69.8 ± 6 a	44,567.5 ± 1.77.7 a	2.5 ± 0.1 a

Values are means ± SEM of 6 replicates per treatment. Different letters in the same column and for the same crop indicate significant differences at *p* < 0.05 (Tukey HSD test).

**Table 5:** Carbohydrate, protein, polyphenol, nitrate and proline content in edible leaves.

Treatments	Carbohydrates	Proteins	Polyphenols	Nitrates	Proline
	%	mg g^−1^ (DW)	mg g^−1^ (FW)	mg g^−1^ (FW)	µmol g^−1^ (FW)
LC	71.4 ± 5.9	0.5 ± 0.03	0.3 ± 0.02 b	1670 ± 331 b	2.73 ± 0.51
LS	54.1 ± 4.6	0.7 ± 0.1	0.3 ± 0.02 ab	3659 ± 186 a	5.06 ± 0.66
L Intercrop	56.5 ± 4.2	0.6 ± 0.02	0.4 ± 0.03 a	4693 ± 452 a	7.32 ± 2.31
SC	31.6 ± 1.2 b	0.4 ± 0.04 b	0.7 ± 0.05 a	6859 ± 742 a	2.21 ± 0.14
SS	39.4 ± 2.7 a	0.5 ± 0.02 a	0.6 ± 0.04 b	5121 ± 421 ab	2.79 ± 0.22

Values are means ± SEM of 6 replicates per treatment. Different letters in the same column and for the same crop indicate a significant difference at *p* < 0.5 (Tukey’s test for *L. sativa* and unpaired *t*-test for *S. soda*).

## Data Availability

All available data are contained within the article.
